# Obituary—John J. R. Patrick, D.D.S., Belleville, Ills.

**Published:** 1895-09

**Authors:** 


### OBITUARY.

### John J. R. Patrick, D.D.S., Belleville, Ills.

JOHN J. R. PATRICK, D.D.S., died April 10th, at 1 o'clock, of heart disease, aged sixty-nine years. His demise was not unexpected, he having been a confirmed invalid since last August.

The deceased was a native of England, and was born in Liverpool in 1826. He passed his boyhood days in Belfast, Ireland, and when about twenty years of age came with his parents to America. The family lived for a time in Iowa, and later came to St. Louis, where the deceased worked as a silversmith. During his leisure hours he took up the study of dentistry, and soon qualified himself for admission into the ranks of the profession. He then gave up his trade of jeweler and silversmith and devoted himself entirely to his chosen profession.

He came to Belleville early in the fifties, and opening an office began the practice of dentistry and was very successful.

In the fall of 1862, he enlisted in the 130th Illinois (Infantry)

Regiment, of which Judge N. Niles was Colonel, and was Captain of Company G until mustered out in the Spring of 1865.

Dr. Patrick was twice married. His first wife whom he married in England, having died in Belleville about five years ago. His second marriage occurred about three weeks ago with Miss Anna Rischar, of this city, who survives him. He also leaves a niece, Mrs. Marcellas Clouse, of Birkner Station, and two nephews, William and Andrew Boatman, the latter in the State of Washington.

He was a learned paleontologist and had recently completed the second of his two reports on prehistoric skulls. This work was done as curator of the American Dental Association, of which he was a distinguished member.

His very large collection of Indian antiquities, skulls, etc., was sold by him to the Missouri Historical Society.

He was skilled in his profession and delivered special lectures to the students of the Missouri Dental College and State University of Iowa.

He was a member of Hecker Post No. 443, G. A. R.

Dr. Patrick was esteemed outside of his profession as a contributor to the archives of the Smithsonian Institution. His work on prehistoric crania, and exploration of Indian mounds is too well known to need special comment here.

Living as he did in close proximity to St. Louis, he was a frequent visitor to that city as well as a member of various societies in Missouri.

Dr. Patrick was a genial man, friendly in his attitude toward younger men. He was a great student and an omnivorous reader. He was an inventor, an investigator, a controversialist and a well-read physician as well as a dentist. He will be long remembered for his picturesque personality and his earnestness in debate before learned societies. The profession in Illinois will mourn his decease, as in his removal from the active sphere we lose a conspicuous figure from our midst, one who was ever progressive. He did not dwell on the past, it was the future, now alas! no future in this vale of leave.—*Dental Review.*

